# Seven DNA Methylation Biomarker Prediction Models for Monitoring the Malignant Progression From Advanced Adenoma to Colorectal Cancer

**DOI:** 10.3389/fonc.2022.827811

**Published:** 2022-05-12

**Authors:** Wei Wang, Xuecong Zhang, Xiaohui Zhu, Wenzhi Cui, Danli Ye, Guihui Tong, Dingpeng Huang, Juan Zhou, Xuwen Lai, Guangning Yan, Xia Li, Jianbing Fan, Hongwu Zhu, Chengyong Lei

**Affiliations:** ^1^Department of Pathology, General Hospital of Southern Theater Command, People’s Liberation Army of China, Guangzhou, China; ^2^Department of Bioinformatics, School of Basic Medicine, Southern Medical University, Guangzhou, China; ^3^Department of Pathology, Nanfang Hospital and Basic Medical College, Southern Medical University, Guangzhou, China; ^4^Guangdong Province Key Laboratory of Molecular Tumor Pathology, Guangzhou, China; ^5^Department of Gastroenterology, General Hospital of Southern Theater Command, People’s Liberation Army of China, Guangzhou, China; ^6^Department of Oncology, General Hospital of Southern Theater Command, People’s Liberation Army of China, Guangzhou, China; ^7^AnchorDx Medical Co., Ltd., Guangzhou, China; ^8^Department of Urology, Nanfang Hospital, Southern Medical University, Guangzhou, China

**Keywords:** cell-free DNA, advanced adenoma, colorectal cancer, monitoring, methylation model

## Abstract

Advanced adenoma (AA) holds a significantly increased risk for progression to colorectal cancer (CRC), and we developed a noninvasive DNA methylation prediction model to monitor the risk of AA progression to CRC. We analyzed the differential methylation markers between 53 normal mucosa and 138 CRC tissues, as well as those in cfDNA (cell-free DNA) between 59 AA and 68 early-stage CRC patients. We screened the overlapping markers between tissue DNA and cfDNA for model variables and optimized the selected variables. Then, we established a cfDNA methylation prediction model (SDMBP model) containing seven methylation markers that can effectively discriminate early-stage CRC and AA in the training and validation cohorts, and the AUC (area under the curve) reached 0.979 and 0.918, respectively. Our model also reached high precision (AUC=0.938) in detecting advanced CRC (stage III/IV) and presented better performance than serum CEA and CA199 in screening CRC. The cd-score of the SDMBP model could also robustly predict the TNM stage of CRC. Overall, our SDMBP model can monitor the malignant progression from AA to CRC, and may provide a noninvasive monitoring method for high-risk populations with AA.

## Introduction

Colorectal cancer (CRC) is the second leading cause of cancer-related deaths worldwide (1). The adenoma-cancer sequence is the main pathway for most sporadic CRCs. Individuals with advanced adenomas (AAs, size ≥1 cm, high-grade dysplasia, or villous or tubule-villous histology) have a double risk of progression to CRC (2, 3). Colonoscopy may be the best method for the early visual detection and screening of CRC (4–6). However, its invasiveness, time-consuming nature, requirement for bowel preparation and associated high-cost limit its wide application for routine screening of CRC in high-risk populations (7, 8). Quantification of the level of serum carcinoembryonic antigen (CEA) has previously been considered a specific and noninvasive method for identifying occult CRC. However, the low sensitivity (40 to 60%) of this method limits its use. Combining the quantification of both CEA and carbohydrate antigen 199 (CA199) levels can improve the sensitivity of this assessment, but the effect is still limited (9–11). Noninvasive monitoring of patients with AA is key to the early diagnosis and prevention of CRC. Thus, there is an urgent need for specific, sensitive, and noninvasive biomarkers for the early detection of CRC.

Alterations in DNA methylation patterns might represent detectable neoplastic changes related to tumorigenesis (12). Many methylation markers have emerged as useful diagnostic and prognostic biomarkers for CRC (13–16). However, almost all studies on the early diagnosis and screening of CRC have classified patients with CRC and adenoma as affected populations and distinguished them from normal controls (17–21). Few studies have focused on monitoring the risk of AA progression to CRC. In addition, the ctDNA concentration is lower in plasma from patients with early-stage tumors than in plasma from patients with advanced tumors (22). Many diagnostic models based on ctDNA methylation markers for the early screening of solid tumors show superior positivity in advanced tumors, but the sensitivity of these models decreases significantly for early tumors (14, 19, 23, 24).

In this study, we developed a novel methylation diagnostic model and analysis method to achieve sensitive and noninvasive surveillance of high-risk populations with AA progression to CRC. We selected the differentially methylated markers that differed between AA and early-stage CRC (instead of all stages of CRC) at the plasma level for model construction and validation. We further validated the accuracy and robustness of our model in an independent early-stage CRC cohort as well as another advanced CRC cohort. The model demonstrated good predictive performance in both datasets. Therefore, our study may provide a useful model for monitoring the malignant progression from AA to CRC and a new method for monitoring high-risk populations with AA.

## Materials and Methods

### Patient Enrollment and Sample Collection

A total of 237 formalin-fixed paraffin-embedded (FFPE) tissues, including 179 CRC tissues and 58 adjacent normal tissues that were derived from the normal mucosa 5 cm away from the primary cancer, were collected from Southern Hospital of Southern Medical University. Plasma was collected from 262 CRC and 98 AA patients at the Southern Hospital of Southern Medical University and General Hospital of Southern Theater Command from November 2015 to October 2019. Blood samples with a 10-mL aliquot were collected from CRC or AA patients 1-3 days before surgery or colonoscopy with acellular DNA BCT tubes (Streck, catalog 218962). Plasma was separated by centrifugation at 1600 rpm for 10 min at 4°C followed by a second centrifugation at 16000 rpm for 10 min at 4°C and stored at -80°C until DNA isolation. The tissue and plasma samples came from different patients. This study was approved by the Ethics Committee of Southern Hospital and General Hospital of Southern Theater Command. No informed consent was required because patient information was desensitized and the data were analyzed anonymously.

To verify the accuracy and reliability of our methylation panel and method, we adopted The Cancer Genome Atlas (TCGA) methylation cohort for validation. Data and clinical characteristics associated with the human methylation 450 K array of colon adenocarcinoma (COAD) were available from TCGA (https://cancergenome.nih.gov/).

Serum CEA and CA199 levels were measured at a local clinical laboratory, and levels of less than 5 μg/mL and 37 U/mL, respectively, were considered within reference ranges (25).

### Isolation of Tissue Genomic DNA and Plasma Cell-Free DNA

The QIAamp DNA FFPE Tissue Kit (Qiagen, Cat# 56404) was used to isolate tissue gDNA from FFPE samples according to the manufacturer’s protocol. cfDNA was isolated from plasma using the Bioo NextPrep-Mag™ cfDNA Isolation Kit (Bioo Scientific, Austin, TX, USA, Cat# NOVA-3825) following the manufacturer’s protocol. The concentration and quality of cfDNA was examined using the Qubit™ dsDNA HS Assay Kit (Thermo Fisher Scientific, Cat# Q32854) and the Agilent High Sensitivity DNA Kit (Agilent, Cat# 5067-4626). cfDNA with a yield greater than 3 ng and without obvious contamination of gDNA was used for further DNA library construction.

### AnchorIRIS™ Targeted Methylation Sequencing

We used the AnchorDx EpiVisio™ Target Enrichment Kit (AnchorDx, Cat# A0UX00031) and methylation panels (AnchorDx PanMet V2) for target enrichment. A total of 1000 ng of DNA containing up to four prehyb libraries was pooled for target enrichment using the AnchorDx PanMet V2 methylation panel. AnchorDx PanMet V2 included 12624 preselected cancer-specific methylation regions. The total size of the genomic regions targeted by the AnchorDx PanMet V2 panel was 733057 bp which covered 55369 CpG sites. We carried out probe hybridization, purification and final PCR amplification according to the protocols. The AnchorIRIS™ prelibrary construction and target enrichment technologies have been previously described in detail (21, 26).

### Sequencing Data Analysis

Enriched libraries were sequenced by the Illumina HiSeqX Ten Sequencing System. Sequencing adapters and 3’ low-quality bases were trimmed from raw sequencing reads using a routine algorithm and then aligned to the C→T in silico converted hg19 reference genome using Bismark version 0.17.0 (Bowtie2 as the default aligner behind Bismark). Aligned reads were further evaluated by Picard (version 2.5.0) to obtain metrics that measured the performance of target capture-based bisulfite sequencing assays (http://broadinstitute.github.io/picard). After the preliminary analysis, we calculated the average coverage as well as the missing rate for each CpG site.

## Statistical Analysis

A differential methylation analysis between normal mucosa and CRC tissues was performed by using the Wilcoxon signed-rank test (P≤.0001) with a mean difference> 0.2. We used the differentially methylated CpG loci (DMLs) to identify the difference in methylated loci between normal mucosa and CRC tissues. The same tests were performed 100 times to identify plasma samples between patients with AA and early-stage CRC by randomly extracting three-quarters of the total samples each time. We selected overlapping methylation markers between tissues and samples to shrink biomarkers and ensure accuracy. Least absolute shrinkage and selection operator (LASSO) regression analysis and random forest in the R package were implemented to select variables and build the diagnostic model using blood samples from the AA and CRC patients in the training cohort. Methylation-correlated blocks (MCBs) have been proven to increase the accuracy of determining allele methylation status. We used our sequencing data to identify MCBs as previously described (14, 27). We also calculated the area under the curve (AUC) to compare the discrimination performances of the model with the serum CEA and CA199 levels. Logistic regression was used to calculate the coefficients of the seven markers in the model, and the formula for calculating the combined diagnostic score (cd-score) was as follows:


Cd−score=(83.610*methylation β value of ZFHX4)  +  (0.3131* methylation β value of ZNF334)+ (14.791* methylation β value of ELOVL2)  +  (109.449* methylation β value of UNC5C)  +  (15.631*methylation β value of LOC146880) + (137.500* methylation β value of SFMBT2) + (44.545*methylation β value of GFRA1) −7.336.

The cutoff value (0.327) was determined by Youden’s index based on the ROC model.

## Results

### Clinical Characteristics of the Study Cohort and Study Flow of Participants

**Figure 1:**
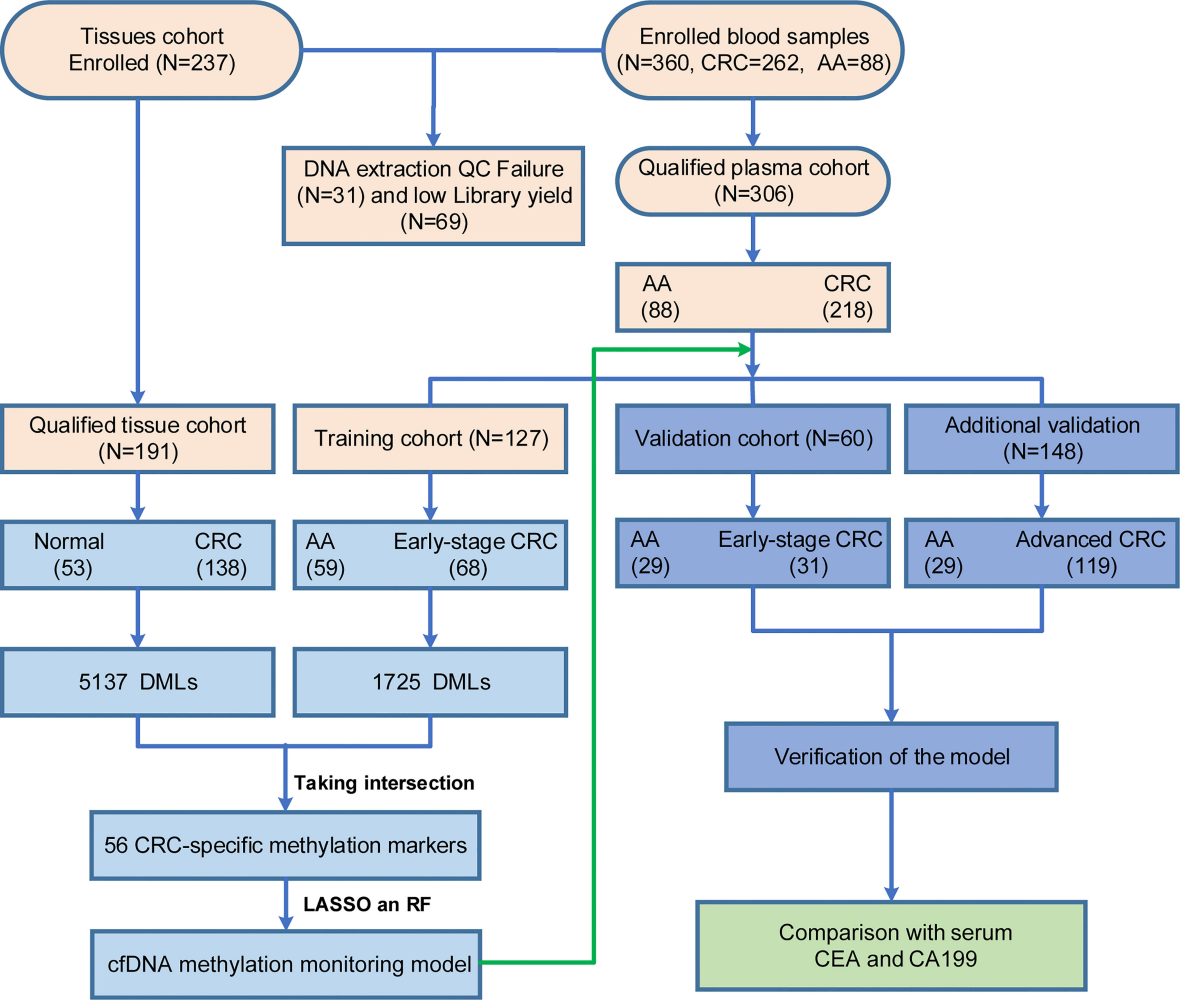
Enrollment of a retrospective study cohort and workflow for building the methylation monitoring model with seven biomarkers. Light orange: quality control of tissues and blood samples; Light blue: construction of the SDMBP model; Purple: verification of the SDMBP model in the training cohort and additional independent cohort; Green: CRC screening performance comparison of the SDMBP model with quantification of the levels of serum CEA and CA199; The validation and additional validation cohort included the same 29 AA patients.

A total of 191 tissues (including 138 CRC tissues and 53 adjacent normal tissues) and 306 blood samples (including 218 CRC patients and 88 AA patients) were collected that passed quality control (QC) and were subsequently subjected to DNA extraction, AnchorIRIS™ library construction and DNA methylation next-generation sequencing (NGS), as shown in **Table 1**. One hundred samples (46 tissues and 54 plasma) were excluded due to DNA extraction QC failure (DNA degradation and contamination; n = 31) or low library yield (n = 69). The 218 CRC patients from whom blood samples were collected included 43 patients with stage I CRC, 56 with stage II CRC, 50 with stage III CRC and 69 with stage IV CRC. The plasma samples from 99 stage I/II CRC patients and 88 AA patients were randomly assigned to the training cohort and validation cohort at a ratio of 2:1. Because the ctDNA concentration and detected methylation signals in early-stage CRC were strikingly weaker than those in advanced CRC, we tried to build a methylation prediction model in early-stage CRC patients and verified it in early-stage CRC patients and patients with advanced disease to improve the model sensitivity for detecting early tumors. Therefore, all advanced CRC (stage III and IV) samples were used as additional validation for the efficiency of the methylation diagnostic model. An overview of the study design is shown in **Figure 1**.

**Table 1:** Clinical characteristics of the qualified tissue and plasma cohort.

Sample	Tissue		Plasma	
Characteristics	Normal	CRC	AA	CRC
Total (n)	53	138	88	218
Gender				
Male	27	85	66	135
Female	26	53	24	83
Age (years)	55(25~68)	58(25~79)	56(32-80)	55(25-83)
≥50	36	103	63	178
<50	17	35	25	40
Stage				
I	NA	27	NA	43
II	NA	30	NA	56
III	NA	33	NA	50
IV(IV_M*)	NA	35 (40**)	NA	69
Tumor site				
Right colon	NA	95	43	171
Left colon	NA	43	18	47
Whole colon	NA	NA	27	0
CEA quantification				
CEA≥5ng/ml	NA	NA	6	75
CEA<5ng/ml	NA	NA	82	143
CA199 quantification				
CA199≥37u/ml	NA	NA	2	48
CA199<37u/ml	NA	NA	86	170

^*^IV_M: Metastatic CRC tissue from stage IV CRC patients; NA, Not Available; **including 27 paired Ⅳ stage tissues and its distant metastasis.

### Identification of CRC-Specific Methylated Markers

**Figure 2:**
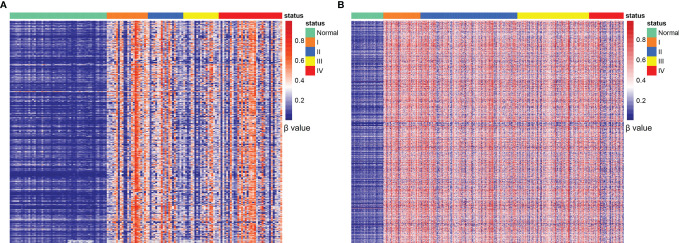
Identification of CRC-specific methylated markers. Unsupervised hierarchical clustering of 5137 DMLs between normal mucosa and CRC tissues **(A)**; 5137 DMLs distributed in normal tissues and stage I-IV CRC samples obtained from the TCGA database **(B)**.

High-throughput AnchorIRIS™ targeted methylation sequencing was performed on 53 normal mucosa and 138 CRC tissues. A total of 5137 DMLs were identified between the normal mucosa and CRC tissues through differential methylation analysis (**Figure 2A**). Unsupervised hierarchical clustering showed that these 5137 DMLs were among the most significantly different DMLs between CRC and normal mucosa in the TCGA cohort (including 285 CRC and 38 normal mucosa samples) (**Figure 2B**). These results indicated that the selected methylation markers (5137 DMLs) were stable, reliable, and specific for distinguishing CRC from normal mucosa. Next, the Wilcoxon signed-rank test was performed 100 times to identify DMLs from plasma between 59 AA and 68 early-stage CRC (stage I and II) patients with a cutoff value of P ≤ 0.01 and mean difference > 0.005. Overall, 1725 DMLs appeared over 80 times out of a total of 100 repetitive tests of plasma between AA and early stage CRC patients. We selected the common shared DMLs between CRC tissues and plasma and obtained 386 overlapping DMLs that were finally assembled into 56 MCBs using a Pearson correlation method with an r^2^ cutoff of 0.5. These 386 DMLs were distributed differently between plasma from AA and CRC patients, as well as between normal mucosa and CRC tissues (**Supplementary Figure 1**).

### The Seven-Methylation-Marker Prediction Model Effectively Monitors the Malignant Transformation of AA and Predicts Early CRC

**Figure 3:**
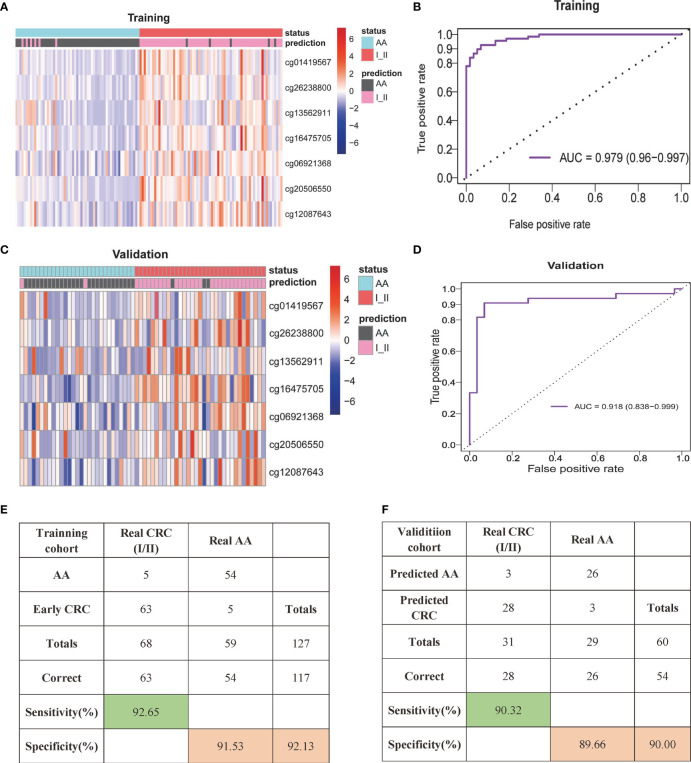
cfDNA methylation analysis for early-stage CRC diagnosis. **(A, B)** Unsupervised hierarchical clustering of the seven selected markers between AA and early stage CRC in the training **(A)** and validation **(B)** cohorts. Each row represents an individual patient, and each column represents a CpG marker. **(C, D)** Receiver operating characteristic (ROC) curve and the related AUCs of the SDMBP model for diagnosing CRC in the training **(C)** and validation **(D)** cohorts. **(E, F)** Confusion matrices built from the SDMBP model in the training **(E)** and validation **(F)** cohorts. The beta values of the DMLs were normalized by the z score method.

LASSO regression analysis using Lambda determined by 10-fold cross-validation was applied for variable selection. Then, the selected variables were used for the next analysis. A random forest model was built to remove variables with minimum feature importance. The remaining variables were also used for model construction, and useless variables were removed using the same method. This process was iterated until optimal variables were identified according to the highest classification accuracy. Here, we selected seven DNA methylation markers: cg01419567 (ZFHX4), cg26238800 (ZNF334), cg13562911 (ELOVL2), cg16475705 (UNC5C), cg06921368 (LOC146880), cg20506550 (SFMBT2) and cg12087643 (GFRA1). Logistic regression was used to calculate the coefficients of the seven markers in the model and develop the formula. We constructed the SDMBP by using the seven MCBs (**Table 2**). Thus, we obtained a cfDNA methylation model classifier that can differentiate AA from early-stage CRC. The SDMBP model presented high precision in both the training and validation cohorts (AUC = 0.979 and AUC = 0.918, as shown in **Figures 3A–D**). This DNA methylation model also achieved a sensitivity of 92.65% and specificity of 91.53% for discriminating early-stage CRC from AA in the training cohort and a sensitivity of 90.32% and specificity of 89.66% in the validation cohort (**Figures 3E, F**). The prediction results suggest that the SDMBP model can distinguish early CRC from AA as well as pathomorphological diagnosis.

**Table 2:** Characteristics of the seven methylation markers and their coefficients in diagnosis.

MCBs	Target ID	Ref Gene	AUC	Coefficents
8-77594526	cg01419567	ZFHX4	0.823	83.610
20-45142116	cg26238800	ZNF334	0.818	109.449
6-11044110	cg13562911	ELOVL2	0.642	14.791
4-96469458	cg16475705	UNC5C	0.847	66.924
17-62775860	cg06921368	LOC146880	0.581	15.631
10-7452563	cg20506550	SFMBT2	0.78	137.500
10-118033370	cg12087643	GFRA1	0.781	44.545

### The SDMBP Model can Accurately Predict Advanced CRC (Stage III/IV)

**Figure 4:**
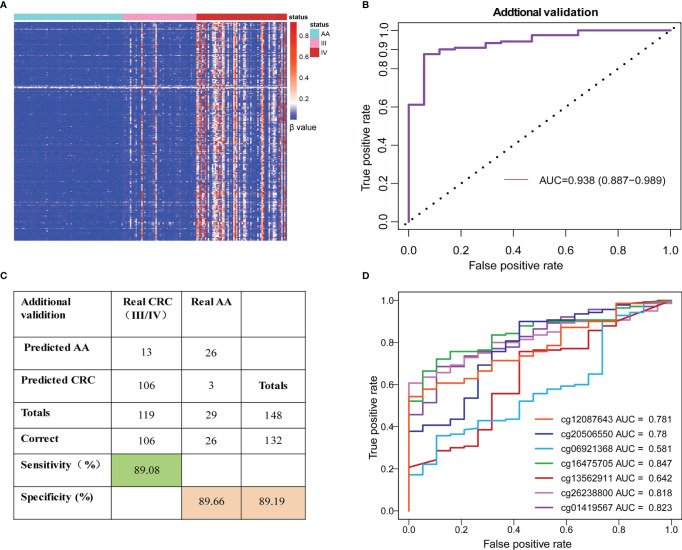
Performance of the SDMBP model in distinguishing AA from advanced CRC. **(A)**Unsupervised hierarchical clustering of 386 overlapping DMLs (equal to 56 MCBs) in cfDNA from patients with AA and stage III/IV CRC. **(B)** ROC curves and the corresponding AUCs of the SDMBP model for diagnosing advanced CRC. **(C)** Confusion matrices built from the model using patients with stage III/IV CRC. **(D)** ROC curves and the corresponding AUCs of diagnostic performance for each methylation marker in the model.

Unsupervised hierarchical clustering showed that the 56 preselected differentially methylated markers (including 386 DMLs) were also located in the most significant region of cfDNA and showed a greater difference between the AA and advanced CRC (stage III/IV) patients (**Figure 4A**). The SDMBP model was highly accurate for predicting early-stage CRC patients. Since advanced CRC patients usually have higher cfDNA and methylated marker levels than early-stage patients, we evaluated the performance of the methylation monitoring model in distinguishing AA from advanced CRC based on blood sample assessment. Because of the limited number of AA patients, we repeatedly used the 29 AA patients in the validation cohort. The SDMBP model displayed good performance in verifying advanced CRC with high sensitivity (89.08%) and specificity (89.66%) (**Figures 4B, C**). We further evaluated the performance of each single methylation marker in distinguishing CRC from AA. The AUCs were 0.823, 0.818, 0.642, 0.847, 0.581, 0.78 and 0.781, respectively (**Figure 4D**). In particular, cg01419567 (ZFHX4), cg26238800 (ZNF334) and cg16475705 (UNC5C) acted more effectively (**Figure 4D**). Overall, the SDMBP model based on the training set of AA and early-stage CRC patients can be used to accurately screen for advanced CRC (stage III/IV).

### The SDMBP Model Is Significantly Better Than Quantifying the Serum CEA and CA199 Levels for CRC Screening

**Figure 5:**
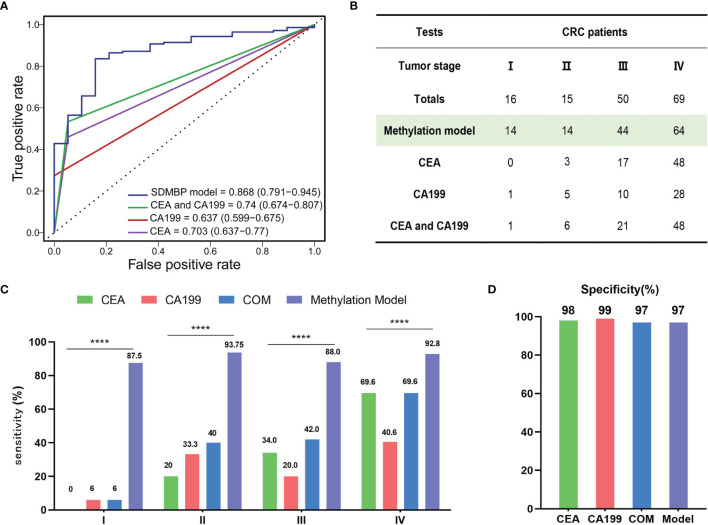
Comparison of the SDMBP model with quantification of the levels of serum CEA and CA199 for diagnosing CRC. **(A)** ROC curves and corresponding AUCs of the SDMBP model, the CEA level, the CA199 level and the combination of the levels of CEA and CA199 for diagnosing CRC in the validation dataset; **(B)** The diagnostic efficiency comparison of the SDMBP model, the CEA level, the CA199 level and the combination of the levels of CEA and CA199 for discriminating AA from CRC of different TNM stages in the validation dataset; **(C, D)** Sensitivity and specificity comparison of the SDMBP model, the CEA level, the CA199 level and the combination of the levels of CEA and CA199 in CRC patients with different TNM stages in the validation dataset. Statistical significance was assessed by the χ2 test **(C)**. ****P < 0.0001.

To compare the performance of the SDMBP model with that of quantifying the levels of serum CEA and CA199, we included 150 CRC patients for further analysis. The SDMBP model demonstrated marked superiority over the level of CEA, the level of CA199 and the combined levels of both markers for screening CRC (as shown in **Figure 5A**), with AUCs of 0.868, 0.703, 0.637 and 0.74, respectively. In particular, none of the stage I CRC patients in validation cohort were identified based on the assessment of the serum CEA level, and only one was identified based on the CA199 level. However, the model predicted 14 of 16 CRC patients (87.5%) with stage I disease. Furthermore, in stage II and III CRC patients, the sensitivity of our methylation model was over fourfold and twofold higher than the assessment of the CEA level (93.75% vs. 20% and 88% vs. 34%) and over twofold higher than assessment of both the CEA and CA199 levels (93.75% vs. 40% and 88% vs. 42%). Even for stage IV CRC patients with a high tumor burden, the SDMBP model showed higher sensitivity than the assessment of the levels of CEA, CA199 and the combination of both (92.8% vs.69.6%, 40.6% and 69.6%) (**Figures 5B, C**). However, there was no significant difference in specificity among the four screening methods (**Figure 5D**). Our comparison results demonstrate that the SDMBP model is more precise than the assessment of the levels of serum CEA and CA199 for CRC screening.

### The Cd-Score of the SDMBP Model Is Significantly Positively Correlated With the TNM Stage of CRC

**Figure 6:**
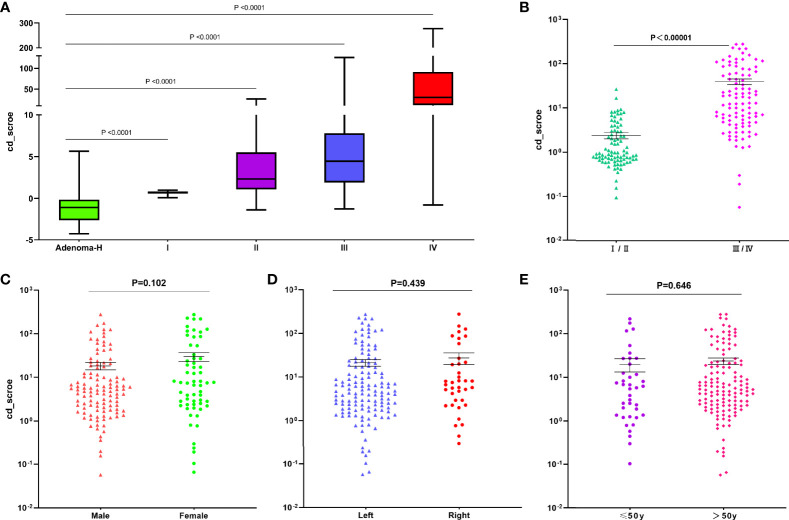
Application of the cd-score of the SDMBP model for predicting tumor stage and different clinical parameters in CRC patients. Cd-score and sensitivity of the SDMBP model in CRC patients with different disease stages (I, II, III and IV) **(A, B)**; Cd-score and sensitivity of the SDMBP model in male patients and female patients **(C)**; in patients with a primary tumor location on the left or right colon **(D)**; and in patients less than 50 years old or over 50 years old **(E)**. Statistical significance was assessed by unpaired t test.

Related research results indicated that the cd-score of the predictive model may be used to classify the severity of the disease (14, 19). Therefore, we further assessed the cd-score (the calculation method is described in the Materials and Methods section) of the SDMBP model for differentiating between AA and CRC. We found that the cd-score could differentiate AA from CRC patients with different TNM stages (**Figure 6A**). These results showed that there was a strong correlation between the cd-score and tumor TNM stage. Patients with early-stage CRC (stage I and II) had substantially lower cd-scores than those with advanced-stage (III and IV) CRC (**Figure 6B**). However, no significant difference existed among other clinical parameters, such as age (older than 50 years and younger), sex (male and female), and tumor location (left and right colon) (**Figures 6C–E**). Therefore, our analysis suggests that the cd-score of our model is significantly positively correlated with the TNM stage of CRC, and may be used as a potential prognostic predictor of CRC.

## Discussion

The adenoma-carcinoma sequence is widely thought to represent the process by which most, if not all, CRCs arise. Importantly, compared with those with ordinary adenoma, patients with AA are at more than double the risk of subsequent CRC (2). Therefore, ongoing periodic surveillance in patients with AA is especially crucial for preventing CRC. Individuals with AA are advised to undergo repeated colonoscopy every 3 years, as well as CEA and CA199 quantification to monitor a lesion and prevent subsequent CRC (28, 29). However, colonoscopy requires a long appointment time and bowel cleansing, is often painful, and can be influenced by bias due to varying observers and experience levels. Quantification of CEA, even in combination with CA199, is also limited by its sensitivity and specificity. This study aimed to discover aberrantly methylated CpG dinucleotides in cfDNA between AA and early-stage CRC samples to monitor the malignant transformation of AA.

Aberrant methylation is a crucial feature of carcinogenesis and usually contributes to the inactivation of gene expression. The evolution from colorectal adenoma to CRC is associated with increasing hypermethylation of CpG islands (CGIs) in the promoter regions of tumor suppressor genes. Therefore, it may be one of the first detectable neoplastic changes associated with tumorigenesis (30, 31). A number of studies have identified some specific DNA methylation sites or DNA methylation profiles, such as SEPT9, as useful biomarkers for the early diagnosis and screening of CRC (18, 23, 32, 33). Xu et al. established a CRC early screening model that classified adenomas and CRC into intestinal neoplasia and distinguished them from healthy samples (14). In fact, almost all studies on CRC screening have constructed diagnostic models based on differences between healthy samples and malignant lesions that contain adenoma and CRC (21, 23, 34, 35). An ideal methylation feature that can differentiate AA from early-stage CRC has not been investigated and could be crucial in CRC surveillance, early detection and prevention. Thus, herein, we built a novel methylation signature that can be used to monitor malignant progression from AA to early stage CRC.

Genome bisulfite sequencing enables the high-throughput detection of large-scale methylation markers. The unique AnchorIRIS™ prelibrary construction and target enrichment techniques allow the high-resolution and high-throughput quantification of multiple CpG sites, even in samples with low methylation frequency. This approach has shown superior performance in the noninvasive diagnosis of early-stage lung cancer and CRC (21, 26, 36) as well as in recurrence monitoring for bladder cancer (37). Therefore, we applied this high-throughput targeted DNA methylation sequencing assay to detect CpG sites and built a methylation model. The ctDNA concentration and detected methylation signal are much lower in plasma from patients with early-stage tumors than in plasma from patients with advanced tumors. Many constructed diagnostic models of solid tumors based on methylation markers in ctDNA showed poorer positivity in early tumors than in advanced patients (14, 19, 26, 37). The novelty of our model and analysis method is that we observed the differences in ctDNA concentration and released methylation signals between early-stage CRC and advanced CRC to improve the sensitivity for monitoring early tumors. Therefore, the differentially methylated markers between AA and early-stage CRC (instead of all stages of CRC) were selected for model construction, avoiding the low sensitivity of detecting early-stage disease because of the methylation signal difference between early-stage CRC and advanced CRC. The constructed methylation model achieved a sensitivity of 90.32% and specificity of 89.66% in detecting stage I and II CRC in the validation cohort, and performed well in distinguishing AA from advanced CRC (stage III/IV) equally, with a sensitivity of 89.08% and a specificity of 89.66%. However, the sensitivities of the use of the levels of CEA, CA199 and both CEA and CA199 were 45.33%, 29.33%, and 50.67%, respectively, in both cohorts. Our model prevails in detecting early-stage CRC because we improved the routine method for model construction.

Noninvasive screening tests emphasize the early detection of stage I and II CRC because this is key to reducing the morbidity and mortality of CRC. The Epi-proColon assay displayed 44.7% sensitivity for detecting stage I and II CRC using methylated SEPT9 in plasma (33). In a recent study using multimarker DNA methylation, the detection rates for stage I and II CRC patients were 64.3% and 81.3%, respectively (34). In a cohort of 2105 individuals, the BCAT1/IKZF1 blood test identified 56% of all early-stage CRCs (stage I and II) (23). The cfDNA methylation biomarker model constructed by Lan exhibited improved sensitivities of 85.9% and 83.7% for identifying stage I and II CRC, respectively (21). Our monitoring model based on seven methylation markers showed a superior sensitivity of 90.32% in distinguishing AA from stage I/II CRC. Therefore, this model may provide a useful tool for monitoring carcinogenesis from AA to CRC. Although the positive cases identified by the SDMBP model require further verification by colonoscopy, the model can reduce the screening times of invasive colonoscopy during follow-up for high-risk AA patients.

Furthermore, each single methylation marker in the model performed well in distinguishing CRC from AA. The AUCs for the seven markers were 0.823, 0.818, 0.642, 0.847, 0.581, 0.78 and 0.781, respectively. Of these, cg01419567 (ZFHX4), cg26238800 (ZNF334) and cg16475705 (UNC5C) exhibited the best performance. UNC5C is a tumor suppressor gene. Aberrant methylation of the UNC5C gene has been proven to be frequently associated with advanced or late-stage CRC (24). ZFHX4 (zinc finger homeobox 4) is a putative transcription factor. A ZFHX4 mutation can apparently decrease the lifetime of CRC patients, implying that ZFHX4 may be a vital factor for prognosis (38). The function of the ZNF334 gene, which encodes a newly described zinc finger protein, is unknown in tumors. With further exploration, these methylation markers may serve as potential targets for cancer diagnosis or treatment.

In conclusion, we developed a model and analysis method that includes seven methylation biomarkers for the noninvasive screening and early detection of the progression of AA to CRC. This novel model achieved greatly superior sensitivity over the quantification of the CEA level, the CA199 level and the combination of both the CEA and CA199 levels. It also greatly improved the detection sensitivity for early-stage CRC compared to the methylation model built by the traditional method. This approach may help reduce the invasiveness, complications and high cost of routine colonoscopy screening for high-risk populations (such as those with AA), making it attractive for use in clinical decision making for a variety of patients and situations. A large-scale, multicenter and prospective clinical trial is needed to further validate the clinical applicability and robustness of this model in China.

## Limitations

Almost all noninvasive CRC screening tests, including various methylation models and fecal immunochemical tests (FITs), screen for intestinal neoplasia, including CRC and adenoma. There is no appropriate noninvasive biomarker to monitor the progression from AA to CRC. Therefore, the performance of the methylation model in distinguishing AA from early CRC can only be compared with that of serum CEA and CA199, which have shown low sensitivity in clinical practice. Hence, the stability and accuracy of our methylation model should be further validated in a prospective, multicenter trial.

The plasma samples, including 99 cases of early stage CRC and 88 cases of AA, were randomly divided into the training set and validation set at a ratio of 2:1, resulting in too few samples in the validation set (60 cases). Therefore, the robustness of the methylation model should be further validated with a larger number of plasma samples.

## Data Availability Statement

The raw data presented in this article are not readily available because China has strict regulations on the management of genetic resources, which can be available from the corresponding author on reasonable request. The clinical and beta value matrix data in this paper have been uploaded onto https://github.com/COADmethylation/EarlyScreen.

## Ethics Statement

The studies involving human participants were reviewed and approved by Ethics Review Board of Nanfang Hospital and early-stage General Hospital of Southern Theater Command. Written informed consent for participation was not required for this study in accordance with the national legislation and the institutional requirements.

## Author Contributions

WW, CL, JF and XHZ designed and perform the study, and wrote the manuscript. XCZ took on the statistical analysis. WW, XCZ and XHZ contributed equally to this work. WC, DY, GT, XWL, GY, XL gave assistance in collecting tissue samples and organizing clinical information. DH, JZ and HZ were responsible for collecting the plasma samples of AA and early stage CRC and managing patients. All authors were involved in writing the paper and had final approval of the submitted and published versions.

## Conflict of Interest

JF and XL are employees of AnchorDx Medical Co., Ltd. or AnchorDx, Inc.

The remaining authors declare that the research was conducted in the absence of any commercial or financial relationships that could be construed as a potential conflict of interest.

## Publisher’s Note

All claims expressed in this article are solely those of the authors and do not necessarily represent those of their affiliated organizations, or those of the publisher, the editors and the reviewers. Any product that may be evaluated in this article, or claim that may be made by its manufacturer, is not guaranteed or endorsed by the publisher.

## Supplementary Material

Supplementary Figure 1 Characterization of the overlapped distribution of 386 DMLs in tissues and plasma. The identified 386 DMLs were distributed differently between plasma from AA and CRC patients as well as between normal mucosa and CRC tissues.

## Abbreviations

AA, advanced adenoma; ROC, receiver operating characteristic; AUC, area under curve; CA199, carbohydrate antigen 199; CEA, carcino-embryonic antigen; cfDNA, cell-free DNA; CpG, cytosine-phosphoric-guanine; DML, differentially methylated CpG loci; CRC, colorectal cancer; ctDNA, circulating tumor DNA; TCGA, the Cancer Genome Atlas; MCBs, Methylation-correlated blocks; FFPE, formalin-fixed paraffin-embedded; FIT, fecal immunological test; NGS, next-generation sequencing; QC, quality control; LASSO, Least Absolute Shrinkage and Selection Operator; RF, random forest.
