# Supplementary figures and images for: Seven DNA Methylation Biomarker Prediction Models for Monitoring the Malignant Progression From Advanced Adenoma to Colorectal Cancer
Source: Front Oncol. 2022 May 12;12:827811. doi: 10.3389/fonc.2022.827811 (PMC9133334; doi:10.3389/fonc.2022.827811)

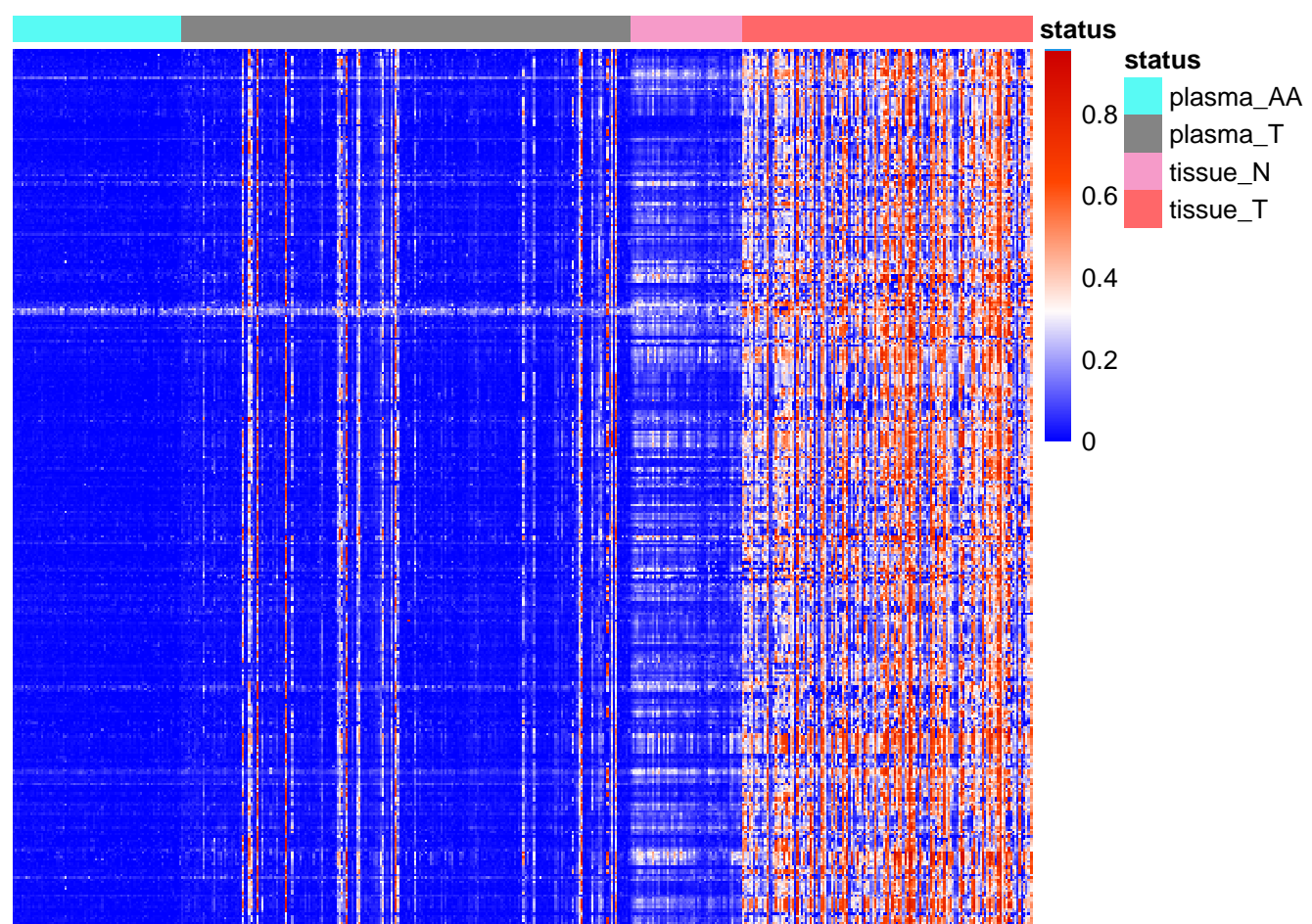

Supplement: Supplementary Figure 1 — Characterization of the overlapped distribution of 386 DMLs in tissues and plasma. The identified 386 DMLs were distributed differently between plasma from AA and CRC patients as well as between normal mucosa and CRC tissues. [file Image_1.pdf]
